# Prediction of Smartphone Addiction Among Korean Adolescents Based on Physical Activity and Mental Health: A Machine Learning Analysis Using LASSO and SHAP From the Korea Youth Risk Behavior Survey

**DOI:** 10.31083/AP46201

**Published:** 2026-02-26

**Authors:** Kihyuk Lee, Wooin Seo, Se Young Jung

**Affiliations:** ^1^Office of Hospital Information, Seoul National University Bundang Hospital, 13605 Seongnam, Republic of Korea; ^2^Department of Family Medicine, Seoul National University Bundang Hospital, 13620 Seongnam, Republic of Korea; ^3^Department of Family Medicine, College of Medicine, Seoul National University, 03080 Seoul, Republic of Korea

**Keywords:** smartphone addiction, adolescents, physical activity, mental health, SHapley Additive exPlanations (SHAP), Least Absolute Shrinkage and Selection Operator (LASSO), machine learning

## Abstract

**Background:**

Adolescent smartphone overuse is associated with physical inactivity and mental health problems, such as anxiety. However, few studies have analyzed these factors jointly using both linear and non-linear methods. This study aimed to predict smartphone addiction using physical activity and mental health indicators from the 2020 and 2023 Korea Youth Risk Behavior Survey, applying Least Absolute Shrinkage and Selection Operator (LASSO), multiple machine learning models, and SHapley Additive exPlanations (SHAP) analysis.

**Methods:**

A total of 86,744 adolescents were classified into general (n = 63,963), potential risk (n = 20,383), and high-risk (n = 2398) smartphone user groups. For the binary classification, general users were compared with combined-risk users. Twelve key predictors were selected using LASSO. Logistic Regression, Random Forest, Extreme Gradient Boosting (XGBoost), and Light Gradient Boosting Machine (LightGBM) models were implemented with Synthetic Minority Over-sampling Technique balancing; SHAP was used to compare variable importance across models.

**Results:**

LASSO identified moderate physical activity (β = –0.156), strength physical activity (–0.149), loneliness (0.144), smartphone usage time (0.085), and anxiety (0.078) as major predictors. Random Forest and Logistic Regression showed the best recall (0.63 and 0.60); LightGBM had the highest accuracy (0.726). It also achieved the highest Area Under the Receiver Operating Characteristic Curve (AUROC) (0.7108); XGBoost showed the lowest AUROC (0.5621). SHAP consistently ranked anxiety and smartphone usage time as the top predictors, with sleep and physical activity showing variable importance.

**Conclusions:**

Anxiety and smartphone usage time were consistently dominant predictors. Physical activity variables contributed in some models but showed inconsistent importance. These findings highlight the central role of mental health, with behavioral factors playing a secondary, model-specific role.

## Main Points

(1) Smartphone addiction among Korean adolescents is closely associated with mental health factors, particularly anxiety and loneliness, which were consistently identified as key contributors.

(2) Physical activity–related factors were also associated with smartphone addiction; however, their relative influence was limited compared with mental health factors.

(3) This study clarifies the structure of core risk factors underlying adolescent smartphone addiction through an interpretable analytical approach.

## 1. Introduction

With the rise of digital technology, smartphones have become essential tools for daily life, with their frequency of use and people’s level of dependence on them increasing rapidly, particularly among adolescents [1]. However, excessive smartphone use can lead to a range of negative outcomes, including reduced concentration, sleep disturbances, decreased physical activity (PA), and increased anxiety and depression. When such use reaches the level of addiction, it can adversely affect both mental and physical health as well as academic achievement [2, 3, 4]. Consequently, smartphone addiction is increasingly recognized as a public health issue, rather than merely a matter of individual behavior [5].

Key factors associated with smartphone addiction include mental health indicators such as stress, depression, loneliness, and anxiety, as well as sleep satisfaction, PA, and socioeconomic status [6, 7, 8, 9, 10]. In developing the Smartphone Addiction Scale, Kwon *et al*. (2013) [11] identified usage time, withdrawal symptoms, and disruption of daily life as important predictive indicators. Additionally, Kim and Lee (2022) [12] found through Logistic Regression analysis that adolescents with higher levels of smartphone overdependence were significantly more likely to experience higher stress levels and lower sleep satisfaction. Recent studies have further substantiated these psychosocial pathways. In college populations, depression predicted smartphone addiction via the mediating role of emotional exhaustion, underscoring affect-dysregulation mechanisms [13]. In adolescent cohorts, a cross-lagged panel analysis revealed a bidirectional cycle between family dysfunction and Internet addiction over time, highlighting the salience of family context in problematic technology use [14].

Previous studies suggest that insufficient PA may contribute not only to physical health problems but also to smartphone overuse by reducing opportunities for outdoor engagement, physical fatigue, and structured daily routines [15, 16]. However, few studies have systematically examined the predictive value of PA along with mental health factors in adolescent smartphone addiction. Most studies have relied on traditional statistical approaches, which often fail to address complex variable interactions or capture non-linear relationships. To overcome these limitations, this study employed machine learning algorithms that can flexibly capture non-linear and interaction effects, combined with SHapley Additive exPlanations (SHAP)-based interpretability, to enhance transparency in identifying key predictors.

Machine learning-based predictive models have recently been used to address the limitations of traditional statistical analyses. One study applied the Extreme Gradient Boosting (XGBoost) model along with SHAP analysis to predict smartphone addiction with high precision (87.6%) and identified the influence of content-based usage patterns, such as gaming, web-based comics (webtoons), and ebooks [17]. Another study used the Random Forest algorithm to predict smartphone addiction among 2203 adolescents with depressive symptoms (accuracy: 77.4%); SHAP analysis revealed that emotion-focused coping, rumination, and school bullying were major predictors [18]. Although these studies have advanced methodological understanding and contributed to identifying key psychological and behavioral factors, most were conducted with relatively small samples or a limited range of variables, resulting in certain constraints in analytic scope. Accordingly, this study sought to extend previous research by incorporating a broader set of predictors, including mental health, sleep, and PA, within a machine learning framework.

Among the diverse factors linked to smartphone addiction, mental health and PA are particularly salient in adolescence, as they directly influence emotional regulation, lifestyle balance, and overall well-being. Mental health problems such as anxiety, stress, and loneliness have been consistently identified as key psychological predictors of problematic smartphone use [8, 19, 20]. Conversely, regular PA and sufficient sleep have shown protective effects, mitigating the risk of smartphone addiction and related behavioral problems [12, 21]. However, despite the increasing attention being paid to these domains, the complex mechanisms through which mental health, sleep, and PA interact to influence smartphone addiction remain insufficiently understood.

Complementing these gaps, recent adolescent studies have emphasized health behaviors: passive-sensing research has linked higher smartphone use with shorter sleep duration or poorer sleep quality and lower PA [22]; observational data has shown that bedtime procrastination mediates the problematic smartphone use–sleep quality association [23]; and PA has been shown to be inversely related to mobile-phone dependence and may be a protective factor in interventions [24, 25]. However, the multidimensional mechanisms underlying smartphone addiction remain underexplored.

Recent studies on adolescent smartphone use have increasingly emphasized the importance of health behavior factors. Sensor-based (passive-sensing) research has shown that greater smartphone use is associated with shorter sleep duration and lower PA levels [22]; observational data have demonstrated that bedtime procrastination mediates the relationship between problematic smartphone use and sleep quality [23]; and PA has been shown to be inversely related to mobile-phone dependence and may act as a protective factor in behavioral interventions [24, 25]. Despite these findings, few studies have integrated both mental health and PA factors into predictive modeling frameworks to explain adolescent smartphone addiction in a multidimensional manner.

Accordingly, this study aimed to predict the risk of smartphone addiction among Korean adolescents and explore the key contributing factors from multiple perspectives. To this end, Least Absolute Shrinkage and Selection Operator (LASSO) regression was first applied to select the major predictive variables, followed by a comparison of the classification performances of various machine learning models. SHAP analysis was then conducted to visualize the contribution of each variable. Furthermore, by comparing the results of the linear variable selection and non-linear model interpretations, this study sought to identify the influential factors in a multidimensional manner. Based on these analyses, this study aimed to provide empirical evidence to inform future prevention and early intervention strategies.

## 2. Materials and Methods

### 2.1 Participants and Data Collection Procedures

This study was a secondary analysis utilizing raw data from the Korea Youth Risk Behavior Survey (KYRBS, https://www.kdca.go.kr/yhs/) conducted annually since 2005 by the Korea Disease Control and Prevention Agency. The KYRBS is a self-administered online survey targeting middle and high school students nationwide; it collects comprehensive information on adolescents’ health behaviors, mental health, PA, sleep, lifestyle habits, accidents, and addiction-related behaviors. The KYRBS employs a stratified multistage cluster sampling method to ensure national representativeness, considering region and school type (middle, general high, and vocational high schools). Schools were selected as primary sampling units, with classes within those schools randomly selected as secondary sampling units.

**Fig. 1.**
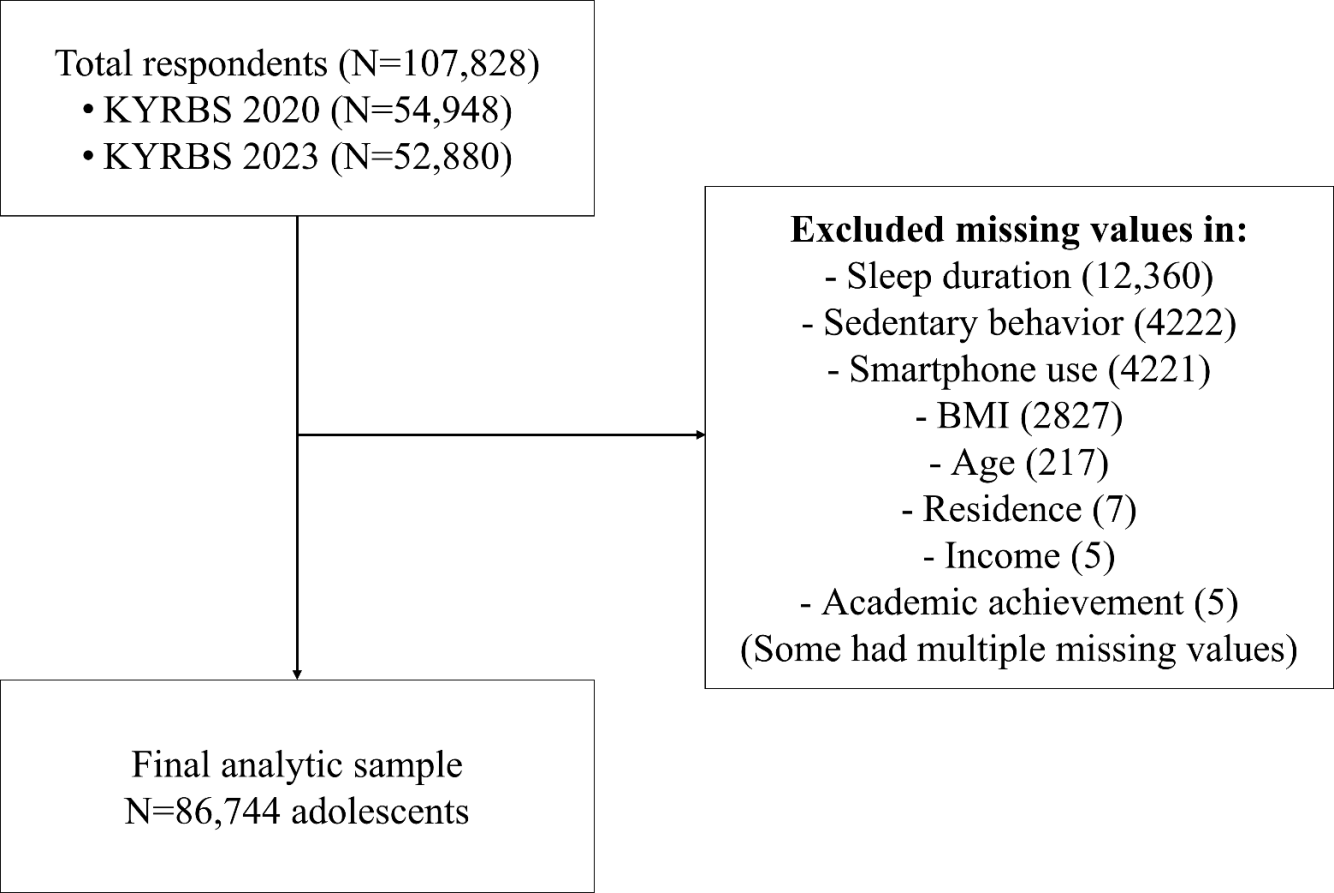
**Flow chart diagram for participant inclusion/exclusion**. KYRBS, Korea Youth Risk Behavior Survey; BMI, body mass index.

This study used data from the 2020 and 2023 surveys, which included items related to smartphone addiction. The 2020 survey was conducted from August 3 to November 13, collecting responses from 54,948 students. The 2023 survey was conducted from August 28 to October 19, with 52,880 participants. After excluding responses with missing values in key variables such as sleep duration (12,360 cases), duration of sedentary behavior (4222 cases), smartphone usage time (4221 cases), body mass index (BMI) (2827 cases), age (217 cases), residence (7 cases), house income (5 cases), and level of academic achievement (5 cases) a final sample of 86,744 adolescents was included in the analysis. Some participants had missing data for multiple items. The participant inclusion and exclusion process is illustrated in Fig. 1.

The data were obtained from the official KYRBS website after research registration. As this study involved a secondary analysis of publicly available data that did not contain personally identifiable information, it was exempt from review by the Institutional Review Board (IRB). The exemption was approved by the Institutional Review Board of Seoul National University Bundang Hospital (approval No. X-2508-992-901).

### 2.2 Classification of Smartphone Addiction Risk Groups

In this study, the level of smartphone addiction was defined based on previous studies and the diagnostic criteria for smartphone overdependence provided by the National Information Society Agency (NIA) of Korea [11, 12]. The scale used in the survey consists of 10 items related to smartphone overdependence, covering three sub-domains: self-control failure, salience (preoccupation and immersion), and serious consequences. Participants responded to each item on a 4-point Likert scale, and total scores were calculated by summing the item responses. The internal consistency of the 10-item smartphone addiction scale was excellent in this study (Cronbach’s α = 0.905, 95% CI = 0.904–0.906).

Based on the NIA’s classification criteria, participants were categorized into three groups: general users (n = 63,963), potential risk users (n = 20,383), and high-risk users (n = 2398). For predictive modeling and to enhance the analytic efficiency and model stability, a binary classification approach was adopted. General users were coded as 0 (non-risk group), whereas potential risk and high-risk users were combined and coded as 1 (at-risk group).

This binary framework followed the recommended best practices for predictive modeling to improve analytical efficiency and model interpretability [26]. Simplifying the outcome variable into two categories helps reduce model variance and enhances stability, particularly with moderately imbalanced data. Similar binary classification approaches have been successfully applied in previous studies to predict problematic smartphone use based on psychological features [27].

### 2.3 Study Variables and Data Preparation

In this study, candidate predictor variables were selected based on prior research and theoretical rationale, focusing on behavioral, psychological, and lifestyle factors related to smartphone addiction. These domains have been consistently identified as key predictors of problematic smartphone use in adolescents, providing a theoretical basis for including variables related to mental health, sleep, and PA [8, 12, 19, 21]. A total of 22 variables were initially considered, and 12 were ultimately selected through LASSO regression analysis.

The candidate variables included demographic characteristics (age, sex, grade, household income, current living arrangements, and level of achievement), subjective perceptions (perceived health, perceived body image, and perceived happiness), PA-related factors (BMI, moderate PA, vigorous PA, strength PA, and duration of sedentary behaviors), mental health indicators (level of stress, sleep satisfaction, sleep duration, sadness, suicidal ideation, loneliness, and anxiety [the Generalized Anxiety Disorder-7, GAD-7]), and smartphone usage time. The smartphone addiction variable was binarized for analysis according to the NIA’s classification criteria described earlier. General users were coded as 0, whereas potential risk and high-risk users were coded as 1.

Subjective perception variables, including perceived health, body image, and happiness, were measured on a 5-point Likert scale, with higher scores indicating more positive perceptions. Although not standardized scales, these items are part of the KYRBS survey and have been repeatedly used in adolescent studies showing acceptable validity [8, 12]. Among the PA variables, BMI was used in its original form without normalization. Participants were classified as engaging in moderate PA if they exercised five or more days per week, vigorous PA if three or more days per week, and strength PA if three or more days per week. These variables were obtained from the KYRBS, which asked participants to recall the number of days in the past seven days they engaged in each activity type: moderate (≥60 min/day, slight increase in heart rate or breathing), vigorous (≥20 min/day, substantial increase), and strength exercises (e.g., push-ups, sit-ups, weight training). These items have been used repeatedly in national surveys and have shown acceptable validity in previous studies [8, 12]. The duration of sedentary behaviors was calculated based on self-reported average daily sitting time over the past seven days, and sleep duration was computed from reported bedtimes and wake-up times during the same period. Smartphone usage time was calculated by multiplying the number of usage days per week by the average duration per day and converting it to hours. Level of achievement was assessed using a single item on overall academic performance over the past 12 months, rated on a 5-point scale (high, upper-middle, middle, lower-middle, and low).

Mental health variables were constructed as follows: Level of stress was measured on a 5-point scale in response to how frequently participants felt stressed in daily life, and sleep satisfaction was assessed based on whether participants felt that their sleep was sufficient for recovery over the past seven days. Sadness and suicidal ideation were measured using binary (yes/no) responses to questions asking whether participants had experienced prolonged sadness or serious suicidal thoughts within the past 12 months. Loneliness was assessed on a 5-point Likert scale indicating the frequency over the past year. Anxiety was assessed using GAD-7, which consists of seven items rated on a 4-point Likert scale, with the total score treated as a continuous variable. The scale demonstrated good internal consistency (Cronbach’s α = 0.897, 95% CI = 0.897–0.898).

All variables were preprocessed as appropriate for the analysis. Categorical variables were either binarized or transformed using one-hot encoding. For example, sex was coded as 0 for male and 1 for female, and the level of achievement was categorized as high, upper-middle, middle, lower-middle, and low levels. Current living arrangement was also binarized as living with family versus other arrangements. Other variables, including PA participation, were recoded according to predefined criteria.

### 2.4 Statistical Analysis

To examine group differences in characteristics, continuous variables were presented as mean ± standard deviation or median with interquartile range, and categorical variables were presented as frequency and percentage. Group comparisons were conducted using one-way analysis of variance for continuous variables and the chi-square (χ^2^) test for categorical variables. All statistical analyses were conducted in the Google Colaboratory environment (Google LLC, Mountain View, CA, USA) using Python (version 3.11.13; Python Software Foundation, Wilmington, DE, USA) with packages such as pandas (https://pandas.pydata.org), SciPy(https://scipy.org), and statsmodels (https://www.statsmodels.org). Statistical significance was set at *p* < 0.05 for all tests.

### 2.5 LASSO-Based Feature Selection

To train the predictive model for smartphone addiction, the dependent variable was defined as smartphone addiction risk (0 = general users, 1 = potential risk and high-risk users). To reduce the dimensionality of the predictor variables and select key factors, LASSO regression was applied. This technique imposes an L1 penalty on regression coefficients, shrinking irrelevant coefficients to zero and thereby performing variable selection. The analysis was performed using the Logistic Regression CV class from the scikit-learn package (https://scikit-learn.org). All input variables were standardized using StandardScaler, and the following settings were applied: penalty = ‘l1’, solver = ‘saga’, and 10-fold cross-validation (CV = 10). The λ (regularization parameter) was selected as the largest value within one standard error of the minimum cross-validation error.

### 2.6 Implementation of Machine Learning Classification Models

Four machine learning classification models were implemented using the variables selected by the LASSO regression: Random Forest, XGBoost, Light Gradient Boosting Machine (LightGBM), and logistic regression. The entire dataset was split into training (80%) and validation (20%) sets by applying stratified sampling to maintain the class distribution. To address class imbalance, the training data were augmented using the Synthetic Minority Over-sampling Technique (SMOTE), adjusting the proportion of the at-risk group to 70% of the non-risk group (sampling_strategy = 0.7). Model performance was evaluated using accuracy, precision, recall, and F1-score, with particular emphasis on recall (sensitivity) for the high-risk group. Additionally, the classification_report function was used to compare class-specific performance metrics comprehensively.

### 2.7 SHAP-Based Interpretation of Feature Importance

To interpret the model predictions and quantitatively evaluate the contribution of each variable, SHAP analysis was conducted. SHAP is a model-agnostic explanation method based on game theory that quantifies and visualizes the contribution of each predictor to the output of a model. In this study, SHAP summary plots were generated for each model to compare variable importance, and these were contrasted with the coefficient-based rankings from the LASSO regression. This comparison enabled an analysis of both consistency and divergence in variable interpretation between the linear and non-linear models.

## 3. Results

### 3.1 Descriptive Characteristics of Smartphone Addiction Risk Groups

Participants were classified into three groups: general users (n = 63,963), potential risk users (n = 20,383), and high-risk users (n = 2398). Descriptive statistics were calculated for each group. Most variables showed a significant deterioration in health and behavioral indicators as the level of smartphone addiction increased (*p* < 0.001). In particular, clear differences were observed in mental health indicators, such as anxiety scores (measured by GAD-7), loneliness, sadness, and suicidal ideation, as well as in PA levels, sleep satisfaction, and sedentary behavior. The high-risk group included a higher proportion of girls and tended to report lower academic achievement and household income. Overall, perceived stress levels were also higher in this group, although specific types of stress were not distinguished. The median smartphone usage time was 4.5 hours in the general group and 7.0 hours in the high-risk group, indicating a difference of approximately 2.5 hours. Detailed comparisons of continuous and categorical variables by group are presented in Tables 1,2.

**Table 1.** **Comparison of continuous variables by smartphone addiction risk group**.

Variables	Total (n = 86,744)			*p*-value
	General (n = 63,963)	Potential_risk (n = 20,383)	High_risk (n = 2398)	
Age	15.03 ± 1.76	15.18 ± 1.70	15.26 ± 1.68	<0.001
BMI	21.49 ± 3.73	21.29 ± 3.61	21.13 ± 3.57	<0.001
Perceived health	3.90 ± 0.89	3.61 ± 0.90	3.46 ± 1.06	<0.001
Perceived body image	3.15 ± 0.97	3.17 ± 1.02	3.21 ± 1.10	<0.001
Perceived happiness	3.83 ± 0.96	3.51 ± 0.95	3.25 ± 1.12	<0.001
Duration of sedentary behaviors (hr)	9.89 ± 3.80	10.11 ± 3.76	10.38 ± 3.95	<0.001
Level of stress	3.11 ± 0.91	3.41 ± 0.85	3.74 ± 0.93	<0.001
Sleep satisfaction	2.98 ± 1.10	2.68 ± 1.05	2.46 ± 1.19	<0.001
Sleep duration (hr)	7.50 ± 1.77	7.26 ± 1.65	7.03 ± 1.65	<0.001
Loneliness	2.37 ± 1.03	2.81 ± 0.99	3.13 ± 1.14	<0.001
GAD-7	3.33 ± 3.94	5.51 ± 4.64	8.37 ± 6.03	<0.001
Smartphone usage time (hr)	4.50 [3.00–6.50]	5.58 [4.00–7.75]	7.00 [5.25–9.50]	<0.001

GAD-7, Generalized Anxiety Disorder-7.

**Table 2.** **Comparison of categorical variables by smartphone addiction risk group**.

Variables		Total (n = 86,744)			*p*-value
		General (n = 63,963)	Potential_risk (n = 20,383)	High_risk (n = 2398)	
Sex	Male	34,114 (53.33)	8903 (43.68)	857 (35.74)	<0.001
	Female	29,849 (46.67)	11,480 (56.32)	1541 (64.26)	
Grade	7th grade	12,757 (19.94)	3102 (15.22)	295 (12.30)	<0.001
	8th grade	11,160 (17.45)	3551 (17.42)	451 (18.81)	
	9th grade	10,756 (16.82)	3853 (18.90)	478 (19.93)	
	10th grade	10,661 (16.67)	3538 (17.36)	361 (15.05)	
	11th grade	9680 (15.13)	3420 (16.78)	449 (18.72)	
	12th grade	8949 (13.99)	2919 (14.32)	364 (15.18)	
Household income	Low	1110 (1.74)	382 (1.87)	96 (4)	<0.001
	Lower-middle	6099 (9.54)	2519 (12.36)	349 (14.55)	
	Middle	29,958 (46.84)	9801 (48.08)	1067 (44.50)	
	Upper-middle	19,290 (30.16)	5928 (29.08)	637 (26.56)	
	High	7506 (11.73)	1753 (8.60)	249 (10.38)	
Current living arrangement	Boarding or shared housing	259 (0.40)	69 (0.34)	8 (0.33)	<0.001
	Institutional facility	151 (0.24)	31 (0.15)	17 (0.71)	
	Living with relatives	271 (0.42)	70 (0.34)	18 (0.75)	
	Dormitory	2062 (3.22)	547 (2.68)	50 (2.09)	
	Living with family	61,220 (95.71)	19,666 (96.48)	2305 (96.12)	
Level of achievement	Low	6830 (8.60)	2898 (11.55)	722 (21.58)	<0.001
	Lower-middle	17,452 (21.99)	6717 (26.76)	892 (26.66)	
	Middle	24,212 (30.50)	7178 (28.60)	735 (21.97)	
	Upper-middle	20,220 (25.47)	5809 (23.14)	627 (18.74)	
	High	10,663 (13.43)	2498 (9.95)	370 (11.06)	
Moderate PA	No	52,707 (82.40)	17,973 (88.18)	2122 (88.49)	<0.001
	Yes	11,256 (17.60)	2410 (11.82)	276 (11.51)	
Vigorous PA	No	40,022 (62.57)	14,200 (69.67)	1752 (73.06)	<0.001
	Yes	23,941 (37.43)	6183 (30.33)	646 (26.94)	
Strength PA	No	46,861 (73.26)	16,471 (80.81)	1999 (83.36)	<0.001
	Yes	17,102 (26.74)	3912 (19.19)	399 (16.64)	
Sadness	No	50,465 (78.90)	13,723 (67.33)	1272 (53.04)	<0.001
	Yes	13,498 (21.10)	6660 (32.67)	1126 (46.96)	
Suicidal ideation	No	57,991 (90.66)	17,070 (83.75)	1754 (73.14)	<0.001
	Yes	5972 (9.34)	3313 (16.25)	644 (26.86)	

Note: Some variables include missing responses; therefore, category totals may not sum to the group sample size. PA, physical activity.

### 3.2 LASSO-Based Predictor Selection for Smartphone Addiction

According to the results of the LASSO regression analysis, key predictors associated with smartphone addiction levels included moderate PA (β = –0.156), strength PA (β = –0.149), perceived health (β = –0.086), sleep satisfaction (β = –0.062), loneliness (β = 0.144), smartphone usage time (β = 0.085), and GAD-7 (β = 0.078). Furthermore, level of achievement (β = –0.040), BMI (β = –0.006), sleep duration (β = –0.003), vigorous PA (β = –0.029), and perceived happiness (β = –0.006) were also identified as contributing predictors. The detailed coefficient values are presented in Table 3.

**Table 3.** **Variable selection and coefficients from LASSO regression for predicting smartphone addiction**.

Variables	Coefficient (β)	Rank	Selected
Age	–	–	NO
Sex	–	–	NO
Grade	–	–	NO
Household income	–	–	NO
Current living arrangement	–	–	NO
Level of achievement	–0.03971605	8	Yes
Perceived health	–0.08557509	4	Yes
Perceived body image	–	–	NO
Perceived happiness	–0.00626179	10	Yes
BMI	–0.00621073	11	Yes
Moderate PA	–0.15576207	1	Yes
Vigorous PA	–0.02916667	9	Yes
Strength PA	–0.14904170	2	Yes
Duration of sedentary behavior (hour)	–	–	NO
Level of stress	–	–	NO
Sleep satisfaction	–0.06159632	7	Yes
Sleep duration (hour)	–0.00270926	12	Yes
Sadness	–	–	NO
Suicidal ideation	–	–	NO
Loneliness	0.14375674	3	Yes
GAD-7	0.07789392	6	Yes
Smartphone usage time	0.08522390	5	Yes

LASSO, Least Absolute Shrinkage and Selection Operator.

### 3.3 Performance Comparison of Machine Learning Models

The classification performances of the four machine learning models—Logistic Regression, Random Forest, XGBoost, and LightGBM—were compared using 12 LASSO-selected predictors. In overall accuracy, LightGBM (0.7260) and XGBoost (0.7146) were the highest, whereas their Area Under the Receiver Operating Characteristic Curve (AUROC) values were 0.7108 and 0.5621, respectively, indicating that the threshold-independent performance was the strongest for LightGBM and weakest for XGBoost. Logistic Regression showed an AUROC value of 0.6991 and Random Forest one of 0.6554. For the risk group (minority class), recall was highest with Random Forest (0.63) and Logistic Regression (0.60), with the top F1-scores for this class (0.49 and 0.48, respectively). Although LightGBM achieved the best overall accuracy and AUROC, its recall in the at-risk group was relatively low (0.34). Considering the macro-averaged F1 to balance the classes, Logistic Regression, Random Forest, and LightGBM were similar (all 0.61), whereas the weighted F1 favored LightGBM (0.71) and XGBoost (0.70). Thus, when prioritizing sensitivity to identify the risk group, Logistic Regression or Random Forest is preferable; when emphasizing overall discrimination and aggregate prediction, LightGBM (and to a lesser extent, XGBoost) is more suitable. The detailed metrics are shown in Table 4.

**Table 4.** **Comparison of classification performance of machine learning models based on LASSO-selected variables**.

Models	Accuracy	AUROC	Class	Precision	Recall	F1-score
Logistic Regression	0.6538	0.6991	General	0.82	0.67	0.74
			Risk	0.39	0.60	0.48
			Macro avg	0.61	0.64	0.61
			Weighted avg	0.71	0.65	0.67
Random Forest	0.6542	0.6554	General	0.83	0.66	0.74
			Risk	0.40	0.63	0.49
			Macro avg	0.62	0.65	0.61
			Weighted avg	0.72	0.65	0.67
XGBoost	0.7146	0.5621	General	0.79	0.84	0.81
			Risk	0.45	0.35	0.39
			Macro avg	0.62	0.60	0.60
			Weighted avg	0.70	0.71	0.70
LightGBM	0.7260	0.7108	General	0.79	0.86	0.82
			Risk	0.47	0.34	0.39
			Macro avg	0.63	0.60	0.61
			Weighted avg	0.70	0.73	0.71

AUROC, Area Under the Receiver Operating Characteristic Curve; XGBoost, Extreme Gradient Boosting; LightGBM, Light Gradient Boosting Machine.

### 3.4 Comparative Feature Contribution Analysis Using SHAP

**Fig. 2.**
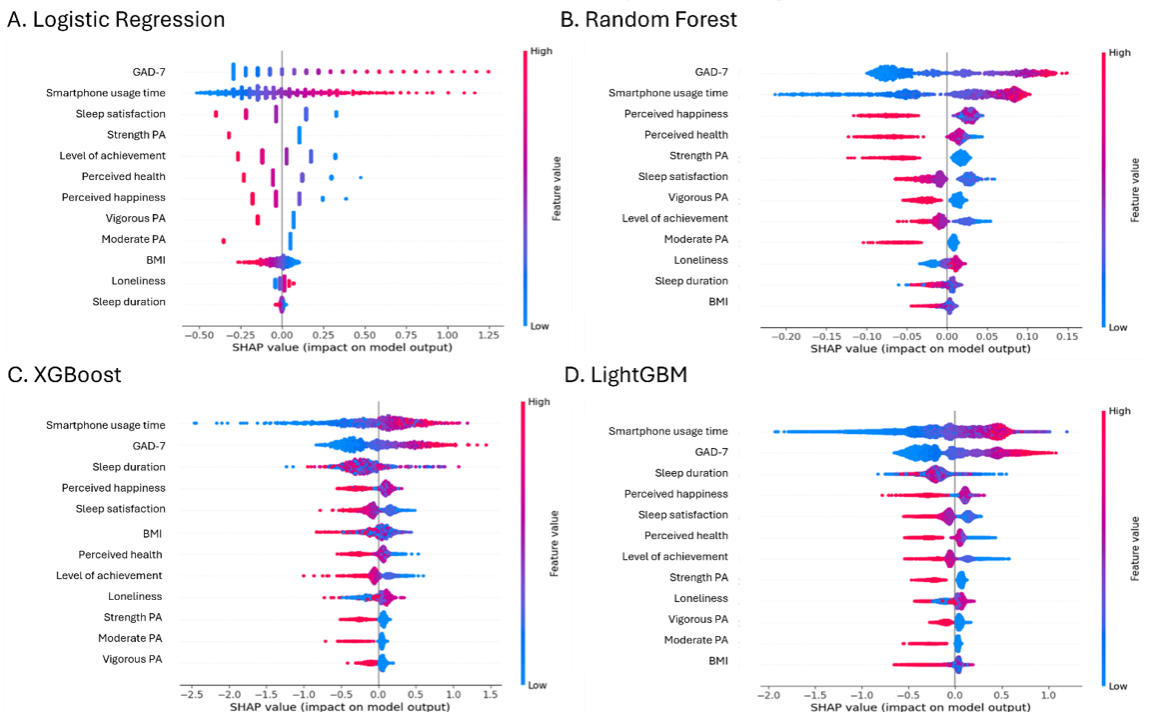
**SHAP analysis of feature importance for predicting smartphone addiction across four machine learning models**. (A) Logistic Regression, (B) Random Forest, (C) XGBoost, and (D) LightGBM. SHAP, SHapley Additive exPlanations.

SHAP analysis was conducted to compare the contribution of each predictor across the machine learning models. In all models, smartphone usage time and GAD-7 consistently emerged as the most important predictors. Moreover, variables such as sleep satisfaction, sleep duration, and perceived happiness ranked high, depending on the model, highlighting the relative importance of sleep-related factors in some models (Fig. 2).

In the Random Forest model, GAD-7, smartphone usage time, perceived happiness, perceived health, and strength PA showed the highest SHAP values. In both LightGBM and XGBoost models, smartphone usage time and GAD-7 were the top contributors, followed by sleep duration, perceived happiness, and sleep satisfaction, indicating that sleep-related factors played a relatively important role in these models. In the Logistic Regression model, GAD-7, smartphone usage time, and sleep satisfaction were the most influential variables, followed by PA-related factors such as strength PA and vigorous PA. Overall, GAD-7 and smartphone usage time consistently ranked among the top two predictors across all four models, whereas the importance of the other variables varied across models.

### 3.5 Comparison of Variable Importance Between Linear and Non-Linear Models

Key predictors of smartphone addiction were identified using two complementary approaches: a variable selection method based on linear regression (LASSO) and SHAP-based interpretation applied to non-linear machine learning models. The importance rankings of the selected variables were then compared across these methods. The analysis revealed that GAD-7 consistently ranked among the top predictors in all machine learning models (first or second place), while ranking sixth in the LASSO regression. This indicates that GAD-7 is a consistently strong predictor across modeling approaches, although its relative importance varies by method.

By contrast, loneliness showed high importance in the LASSO model but ranked relatively lower in non-linear models, reflecting differences in how each method interprets variable contributions. Additionally, sleep satisfaction, moderate PA, and strength PA were commonly identified as key predictors because of their relatively high contributions across multiple models. Some variables with low importance in the LASSO model ranked higher in the non-linear models, underscoring the differences in variable selection and interpretation between the linear and non-linear approaches. The importance rankings of predictors across the different models are shown in Table 5.

**Table 5.** **Comparison of variable importance rankings from LASSO and SHAP across machine learning models**.

Variables	LASSO	Logistic Regression	Random Forest	XGBoost	LightGBM
	Rank	SHAP Rank	SHAP Rank	SHAP Rank	SHAP Rank
GAD-7	6	1	1	2	2
Smartphone usage time	5	2	2	1	1
Sleep duration (hr)	12	12	11	3	3
Sleep satisfaction	7	3	6	5	5
Perceived happiness	10	7	3	4	4
Perceived health	4	6	4	7	6
Strength PA	2	4	5	10	8
Level of achievement	8	5	8	8	7
Vigorous PA	9	8	7	12	10
Moderate PA	1	9	9	11	11
Loneliness	3	11	10	9	9
BMI	11	10	12	6	12

## 4. Discussion

Smartphone addiction is a complex issue influenced by various psychological and behavioral factors, making it difficult to fully explain using a single analytical approach [28]. Therefore, this study targeted Korean adolescents and employed LASSO regression to identify key predictors of smartphone addiction risk. Based on the selected variables, machine learning models and SHAP analysis were applied to visualize the contribution of each factor. Additionally, by comparing the results of LASSO and SHAP, this study analyzed the differences between linear and non-linear approaches and aimed to provide foundational insights for the prevention of smartphone addiction.

Smartphone usage time and anxiety consistently emerged as the most important predictors across all machine learning models [29]. By contrast, loneliness ranked high in the LASSO model but was less prominent in the SHAP-based interpretations. Variables such as sleep satisfaction, sleep duration, perceived happiness, and PA indicators, including moderate PA and strength PA, also demonstrated moderate to high importance depending on the model. Notably, the relative contributions of these factors varied between the linear and non-linear approaches. These findings were consistent with those of previous studies. For instance, Elhai *et al*. (2016) [19] identified anxiety and depression as significant predictors of smartphone addiction, while Demirci *et al*. (2015) [20] reported strong associations between excessive smartphone use, poor sleep quality, and impaired daily functioning. Several studies have also shown that lower PA and higher loneliness are linked to increased smartphone addiction among adolescents and young adults [30, 31]. This relationship was further confirmed by a meta-analysis by Xiao *et al*. (2022) [21], who reported a moderately negative correlation between PA and smartphone addiction (r = –0.243, *p* < 0.001). Moreover, recent studies have extended these associations by clarifying the mechanisms and context: Depression has been linked to smartphone addiction via emotional exhaustion as a mediator [13], and cross-lagged analyses in adolescents have indicated a bidirectional relationship between family dysfunction and problematic Internet use over time [14].

By contrast, a study on university students in rural Turkey reported that smartphone addiction was significantly associated with increased fatigue but showed no statistically significant relationship with PA, sleep satisfaction, or sleep duration [32]. These mixed findings suggest that the factors influencing smartphone addiction may vary depending on regional, cultural, environmental, and demographic characteristics [33]. Nevertheless, the key variables identified in this study, such as smartphone usage time, anxiety, loneliness, sleep satisfaction, and PA, have been consistently reported across various countries and cultural contexts, supporting the external validity and generalizability of our findings.

This study shares certain similarities with, but also differs from, previous studies on smartphone addiction prediction using machine learning [28]. For instance, Kim *et al*. (2024) [17] used XGBoost and SHAP analysis to identify content-related smartphone usage patterns (e.g., gaming, webtoons, and e-books) as key predictors of addiction risk. Dong *et al*. (2025) [34] conducted a longitudinal study on Chinese university students and reported that psychological resilience and the social atmosphere surrounding PA have long-term effects on the levels of smartphone addiction. Osorio *et al*. (2024) [35] examined the relationship between the Big Five personality traits and smartphone addiction in adolescents and compared the predictive performance of several machine learning models, including Random Forest, XGBoost, and LightGBM. Their results showed that the Random Forest algorithm achieved the highest predictive accuracy (89.7%), precision (87.3%), and the highest Area Under the Receiver Operating Characteristic (ROC) Curve (AUC) value. Notably, neuroticism and conscientiousness emerged as the major predictors. However, this study focused primarily on personality traits and did not include behavioral factors, such as PA or sleep, making it difficult to compare the relative importance of diverse predictors.

By contrast, our study used a large-scale, nationally representative sample of 86,744 adolescents and trained a prediction model based solely on self-reported questionnaire items. By applying multiple machine learning techniques alongside SHAP analysis, we visualized the contribution of individual variables, including both mental health (e.g., anxiety and loneliness) and behavioral factors (e.g., smartphone usage time, sleep satisfaction, and PA). This approach distinguishes this study from previous ones and offers practical implications for the development of early screening tools and intervention strategies targeting adolescents [36].

Furthermore, this study employed both LASSO regression and SHAP analysis to examine the consistency between the linear variable selection and interpretations derived from non-linear models. Among the models tested, LightGBM and XGBoost showed the highest overall accuracy (0.726 and 0.715, respectively), whereas Random Forest achieved the highest recall (0.63) for the high-risk group. SHAP analysis showed that smartphone usage time and anxiety were consistently strong contributors across all models, whereas sleep satisfaction, which ranked seventh in LASSO, showed higher importance in SHAP-based models (ranking third to fifth, depending on the model). By contrast, loneliness, which ranked third in LASSO, appeared lower in the SHAP-based rankings (ninth to eleventh), indicating that linear and non-linear methods may capture different aspects of variable importance. Conversely, loneliness, ranked third in LASSO, dropped to ninth to eleventh, suggesting that tree-based models’ non-linear interpretations captured the effects that linear models, such as LASSO, cannot [37]. These pattern differences are also consistent with person- and time-varying evidence: Adolescent trajectory analyses show co-development of problematic smartphone use with depressive symptoms [38], and person-centered work identifies heterogeneous profiles of problematic smartphone use that co-occur with depressive symptoms and vary by self-regulatory characteristics [39, 40]. This suggests that the relationship between certain variables and smartphone addiction may be more complex than a simple linear pattern, supporting the need for multiple analytical approaches. By applying both linear and non-linear techniques, this study was able to confirm the reliability and predictive value of key factors more robustly, thereby contributing not only to model performance assessment but also to the development of interpretable and practically useful AI tools.

Unlike previous studies that focused on a single psychological indicator or specific content type, this study constructed a prediction model based on a more multidimensional and integrated set of factors. Although the recall for the risk group was relatively low despite the application of SMOTE, this limitation highlights the challenge of accurately identifying adolescents at elevated risk based on self-reported behaviors. From a practical perspective, however, models with higher recall remain particularly valuable for early identification in school or clinical settings as improving recall helps ensure that potential cases of problematic smartphone use are not overlooked [36]. Future research should focus on enhancing recall performance by incorporating more detailed behavioral and longitudinal features. Such an approach may offer practical value in informing early warning systems for smartphone addiction among adolescents and guiding the development of tailored intervention strategies, with potential applications in both policy and educational settings.

Despite its strengths, this study had several limitations. First, because the analysis was based on cross-sectional data, it was difficult to draw clear conclusions about the causal relationships between the variables. Future studies should adopt longitudinal designs to examine the temporal sequence of the risk factors for smartphone addiction. Second, as the data were collected through self-reported surveys, the results may have been affected by recall or social desirability bias, potentially reducing the accuracy and reliability of the findings. To enhance objectivity, future research should incorporate more quantitative data sources, such as smartphone usage logs or wearable-based PA measures. Third, although multiple machine learning algorithms were compared, some models (e.g., LightGBM and XGBoost) demonstrated modest recall or AUROC performance, which may affect the precision of risk classification. These results suggest that the current predictive framework should be regarded as an early-stage approach that requires further optimization through parameter tuning and the inclusion of more diverse behavioral and temporal variables. Fourth, this study did not explicitly account for interaction effects between the variables, and the SHAP analysis focused solely on the independent contributions of each factor. Future studies could consider using models that include interaction terms or structural equation modeling to capture more complex relationships [41]. Fifth, because this study targeted adolescents in South Korea, the results may have been influenced by specific cultural and social contexts [33]. Future studies should explore the generalizability of these findings across different cultures and age groups, including those in Western countries, developing regions, and adult or elderly populations. In addition, future research could develop intervention strategies that integrate mental health management and physical activity promotion to reduce the risk of smartphone addiction.

Furthermore, such integrative approaches may contribute to the development of tailored prevention programs that consider both behavioral patterns and psychological characteristics of adolescents.

## 5. Conclusions

Smartphone usage time and anxiety consistently emerged as the most important mental health and behavioral predictors of smartphone addiction risk across all models. By contrast, loneliness ranked high in the LASSO model but was less prominent in the SHAP-based interpretations. Other variables, such as sleep satisfaction, sleep duration, and PA (moderate PA and strength PA), also showed meaningful contributions, although their relative importance varied by model. These findings underscore the complementary nature of the linear and non-linear approaches and enhance the robustness of the identified predictors.

## Availability of Data and Materials

The data used in this study are publicly available from the Korea Youth Risk Behavior Survey (KYRBS) conducted by the Korea Disease Control and Prevention Agency.
